# Traditional Chinese Medicine Monomer Bakuchiol Attenuates the Pathogenicity of *Pseudomonas aeruginosa* via Targeting PqsR

**DOI:** 10.3390/ijms26010243

**Published:** 2024-12-30

**Authors:** Jing Zeng, Xin Ma, Yu Zheng, Dandan Liu, Wanqing Ning, Wei Xiao, Qian Mao, Zhenqing Bai, Renjun Mao, Juanli Cheng, Jinshui Lin

**Affiliations:** 1 Shaanxi Key Laboratory of Research and Utilization of Resource Plants on the Loess Plateau, College of Life Sciences, Yan’an University, Yan’an 716000, China; 17868811824@163.com (J.Z.); ma20230718xin@163.com (X.M.); zy163522@163.com (Y.Z.); 17829553016@163.com (D.L.); 18387120837@163.com (W.N.); 18792388186@163.com (W.X.); 13038581638@163.com (Q.M.); shanxibzq@163.com (Z.B.); mrjnwsuaf@126.com (R.M.); 2 State Key Laboratory for Crop Stress Resistance and High-Efficiency Production, Northwest A&F University (NWAFU), Yangling 712100, China

**Keywords:** *Pseudomonas aeruginosa*, bakuchiol, quorum sensing, virulence

## Abstract

As the antibiotic resistance of pathogens becomes increasingly severe, it is becoming more feasible to use methods that suppress the virulence of pathogens rather than exerting selective pressure on their growth. *Pseudomonas aeruginosa*, a dangerous opportunistic pathogen, infects hosts by producing multiple virulence factors, which are regulated by quorum-sensing (QS) systems, including the *las* systems, *rhl* systems, and *pqs* systems. This study used the chromosome *lacZ* transcription fusion reporter model to screen the traditional Chinese medicine monomer library and found that bakuchiol can effectively inhibit the *pqs* system and related virulence phenotypes of *P. aeruginosa*, including the production of virulence factors (pyocyanin, hydrogen cyanide, elastase, and lectin) and motility (swarming, swimming, and twitching motility) without affecting its growth. Subsequently, through genetic complementation analysis, we found that bakuchiol inhibited the function of the transcriptional activation protein PqsR of the *pqs* system in *P. aeruginosa* in a concentration-dependent manner. Furthermore, molecular dynamics simulation study results indicated that bakuchiol can target PqsR of the *pqs* system, thereby inhibiting the *pqs* system. Among the amino acids in PqsR, ALA-168 may be a key amino acid residue in the hydrophobic interaction between PqsR protein and bakuchiol. Finally, in vivo experiments demonstrated that bakuchiol attenuated the pathogenicity of *P. aeruginosa* to Chinese cabbage (*Brassica pekinensis*) and *Caenorhabditis elegans*. In summary, this study suggests that bakuchiol is an effective inhibitor that targets the *pqs* system of *P. aeruginosa*, providing a new strategy for addressing *P. aeruginosa* infections.

## 1. Introduction

*Pseudomonas aeruginosa* is a common opportunistic pathogen that is widely present in both natural and artificial environments [1]. It is often found in hospitals, and its infection is associated with a high incidence rate and mortality of many diseases, including pneumonia, chronic obstructive pulmonary disease, respiratory infections, burn infections, and cystic fibrosis [2,3,4,5,6,7]. Antibiotic treatment is a common method for eliminating bacterial infections, but this approach is not always successful for *P. aeruginosa* infections [8,9]. *P. aeruginosa* can develop resistance to multiple antibiotics through intrinsic, acquired, and adaptive resistance mechanisms and can easily evade the killing effect of the host immune system [10]. In 2017, *P. aeruginosa* was listed by the World Health Organization as a critical priority pathogen, highlighting the need for the development of new antibiotics and/or drugs to counter infection with this microorganism [11,12]. Given the limited resources of antibiotics for treating *P. aeruginosa* infection, developing new treatment strategies is an urgent task [13].

Quorum-sensing (QS) systems are an important regulatory tool for the survival and infection of *P. aeruginosa* [14]. At present, three classic QS systems (*las*, *rhl*, and *pqs*) have been discovered in *P. aeruginosa* [15,16]. The *las* system and *rhl* system utilize *N*-(3-oxododecanoyl)-l-homoserine lactone (3-oxo-C_12_-HSL) and *N*-butyryl-l-homoserine lactone (C_4_-HSL) as signal molecules, respectively [15,17]. After the signal molecule reaches a threshold, 3-oxo-C_12_-HSL and C_4_-HSL bind to their homologous receptors, LasR protein and RhlR protein [17,18,19], which regulate biofilm formation and the expression of virulence factor-encoding genes, such as exotoxin A, rhamnolipids, siderophores, and alkaline proteases [16,20]. The *pqs* system is the third QS system of *P. aeruginosa*, and it uses 2-heptyl-3-hydroxy-4-quinolone (PQS) or its precursor, 2-heptyl-4-quinolone (HHQ), as the signal molecule [21,22,23]. PQS, the autoinducer, interacts with the transcriptional activator protein PqsR, activating the expression of the PQS synthesis operon, *pqsABCDE,* and regulating the production of elastase, lectin, hydrogen cyanide, and pyocyanin in *P. aeruginosa* [16,24,25]. Previous studies have shown that inhibiting the QS system does not exert pressure on growth, but it does effectively reduce the formation of biofilms and the production of virulence factors in *P. aeruginosa* [26,27,28,29,30,31]. Therefore, developing effective quorum-sensing inhibitors (QSIs) will not only avoid the development of resistance in *P. aeruginosa* but also effectively solve *P. aeruginosa* infection.

*Cullen corylifolium* (Linnaeus) Medikus is a traditional Chinese folk herb that can be used for various diseases, such as psoriasis, menstrual disorders, and vitiligo. Bakuchiol is a monoterpenoid phenolic compound extracted from the seeds of *C. corylifolium* (Linnaeus) Medikusae, with the chemical name 4-(3,7-dimethyl-3-vinyl-octa-1,6-dienyl)-phenol [32]. It also has a wide range of pharmacological activities, including anti-cancer, antioxidant, anti-inflammatory, and lipid-lowering effects [33].

Previous studies have shown that bakuchiol has anti-QS properties [34]. Gene reporter experiments have been conducted on *Escherichia coli* and found that bakuchiol targets the LasR protein. Further molecular investigations in *P. aeruginosa*, such as sedimentation velocity and thermal shift assays, showed that bakuchiol can disrupt the stability of LasR and inhibit its function, ultimately suppressing the production of virulence factors, including pyocyanin, rhamnolipids, and biofilms [34]. By contrast, we constructed a *P. aeruginosa* gene reporter strain and found that bakuchiol can target the PqsR protein. Furthermore, molecular dynamics simulations and site-directed mutagenesis revealed that bakuchiol binds to PqsR and inhibits its transcriptional activity, thereby suppressing the *pqs* system and its related virulence phenotypes (including pyocyanin, hydrogen cyanide, elastase, and lectin). In addition, bakuchiol can reduce the motility and pathogenicity of *P. aeruginosa*. In summary, our study proposes different perspectives on using bakuchiol as a QSI, indicating that bakuchiol can target and inhibit the *pqs* system of *P. aeruginosa*. This provides theoretical guidance for the further development of anti-*P. aeruginosa* drugs based on bakuchiol.

## 2. Results

### 2.1. Bakuchiol Inhibits the pqs System in P. aeruginosa

**Figure 1:**
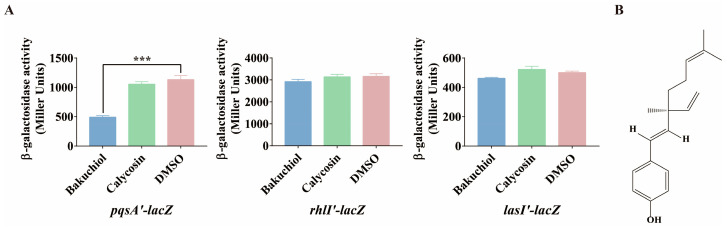
The effect of bakuchiol on gene expression related to quorum-sensing systems. (**A**) Effects of 100 μg/mL of bakuchiol on the expression of QS genes *pqsA*, *rhlI*, and *lasI*. DMSO and calycosin were used as the solvent and Chinese medicine monomer negative controls, respectively. (**B**) The chemical structural formula of bakuchiol. All data represent the results of at least three independent biological replicates. The error bars represent the standard deviations. Student’s *t* test; ***: *p* < 0.001.

In recent years, research on the inhibitory effect of traditional Chinese medicine monomers on the QS systems of *P. aeruginosa* has become an area of focus. In the early stage of this study, a traditional Chinese medicine monomer screening library was established, and preliminary screening was carried out by constructing chromosome lacZ transcription fusion screening models for three QS systems (*pqs* system, *las* system, and *rhl* system). The preliminary screening results showed that bakuchiol has a significant inhibitory effect on the *pqs* system (Table S1). Subsequent analysis used calycosin as a negative control, as both it and bakuchiol are phenolic compounds and calycosin has no effect on the three QS systems (Table S1). Furthermore, the re-screening results showed that compared with DMSO and calycosin (collectively referred to as the control group), the addition of 100 μg/mL bakuchiol reduced the expression level of *pqsA* in *P. aeruginosa*, and this inhibitory effect exceeded 50% (Figure 1A). In addition, compared with *pqsA*, bakuchiol did not have a significant effect on the expression of *rhlI* and *lasI* (Figure 1A). Interestingly, 100 μg/mL of bakuchiol did not affect the growth of *P. aeruginosa* (Table S2 and Figure S1). These results indicated that bakuchiol exerts inhibitory effects on the *pqs* system without causing survival pressure on *P. aeruginosa*, suggesting that bakuchiol can be studied as a potential *pqs* system inhibitor.

### 2.2. The Inhibitory Effect of Bakuchiol on the pqs System of P. aeruginosa Is Concentration-Dependent

**Figure 2:**
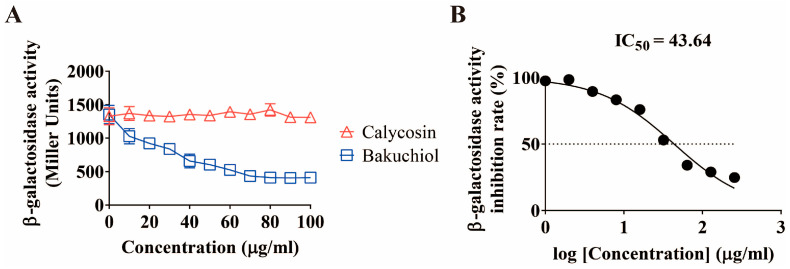
The effect of different concentrations of bakuchiol on the gene expression of *pqsA*. (**A**) Levels of *pqsA* transcription in *Pseudomonas aeruginosa* wild-type strain with increasing bakuchiol concentration. (**B**) The half-maximal inhibitory concentration (IC50) levels of bakuchiol on *pqsA* transcription. The slope of the curve was calculated based on its respective dose–response curves and plotted against the log concentration. All data represent the results of at least three independent biological replicates. The error bars represent the standard deviations.

In this study, we found that when *P. aeruginosa* grew to OD_600_ = 2.0, the inhibitory effect of bakuchiol on *pqsA* reached its maximum (Figure S2). To investigate the effects of different concentrations of bakuchiol on the *pqs* system at OD_600_ = 2.0, we incubated different concentrations (10, 20, 30, 40, 50, 60, 70, 80, 90, 100 μg/mL) of bakuchiol with *pqsA* reporter strains and analyzed the expression of *pqsA*. The results showed that the promoter activity of *pqsA* decreased with increasing concentrations of bakuchiol, and IC_50_ (half-maximal inhibitory concentration) was 43.64 μg/mL (Figure 2). This indicates that the inhibition of *pqsA* by bakuchiol is concentration-dependent, and the inhibition efficiency reached its maximum at 70 μg/mL.

### 2.3. Bakuchiol Targeting the PqsR Protein Inhibits the pqs System in P. aeruginosa

**Figure 3:**
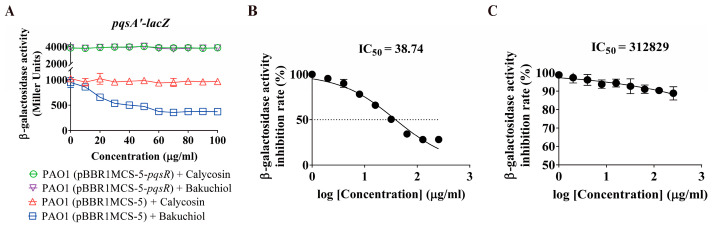
The effect of different concentrations of bakuchiol on the *pqs* system in strains with differential expression of *pqsR*. (**A**) Levels of *pqsA* transcription in strains with differential expression of *pqsR* exposed to increasing concentrations of bakuchiol. (**B**,**C**) The half-maximal inhibitory concentration (IC_50_) levels of *pqsA* transcription in (**B**) PAO1 (pBBR1MCS-5) and (**C**) PAO1 (pBBR1MCS-5-*pqsR*) exposed to various concentrations of bakuchiol. The slope of the curve was calculated based on its respective dose–response curves and plotted against the log concentration. All data represent the results of at least three independent biological replicates. The error bars represent the standard deviations.

The function of the *pqs* system is determined by the biosynthetic gene clusters (*pqsABCDE* and *pqsH*) of the signaling molecule PQS and the PQS receptor protein, PqsR [35]. In the presence of PQS signaling molecules, PqsR will bind to the *pqsA* promoter site, thereby further promoting the synthesis of PQS signaling molecules [36,37]. Therefore, we speculated that bakuchiol may inhibit the expression of *pqsA* through PqsR, ultimately suppressing the *pqs* system. To verify this speculation, we cloned the *pqsR* gene and further overexpressed *pqsR* in a *pqsA* reporter strain. Then, we detected the effect of bakuchiol on the activity of the *pqsA* promoter in the *pqsR*-overexpressing strains. The results showed that in the normal *pqsR* expression strain, as the concentration of bakuchiol gradually increased, the activity of the *pqsA* promoter gradually decreased, and the maximum inhibitory efficiency was achieved at a concentration of 70 μg/mL (Figure 3A). When *pqsR* was overexpressed, the activity of the *pqsA* promoter remained unchanged with increasing concentrations of bakuchiol, and IC_50_ increased from 38.74 to 312829 μg/mL (Figure 3B,C). However, regardless of the normal expression or overexpression of *pqsR*, there was no significant change in the activity of the *pqsA* promoter in the control group. These results confirmed that bakuchiol reduces the expression of the *pqsA* gene by targeting PqsR.

### 2.4. Bakuchiol Binds and Alters the Structure of the PqsR Protein

**Figure 4:**
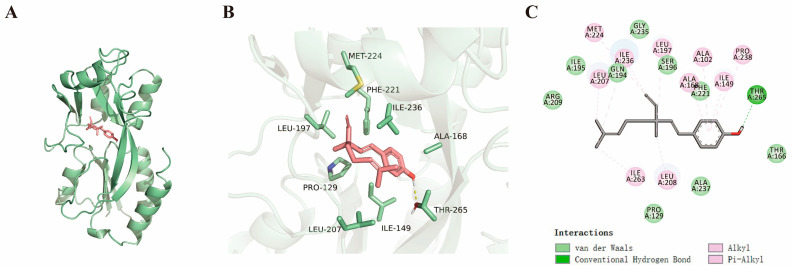
Molecular docking analysis of bakuchiol and PqsR. (**A**) Surface mode diagram of bakuchiol and PqsR. (**B**) Three-dimensional interaction diagram. (**C**) Two-dimensional interaction diagram. The binding energy is −7.5 kcal/mol.

We first used a molecular dynamics simulation study to analyze the binding sites between bakuchiol and the PqsR protein. Figure 4A shows that bakuchiol binds to the central groove of the PqsR protein. Figure 4B,C shows the hydrophobic interactions between bakuchiol and MET-224, LEU-207, LEU-197, ALA-102, ALA-168, ILE-149, PRO-238, ILE-236, PHE-221, and LEU-208 on the PqsR protein. In addition, a hydrogen bond forms between bakuchiol and THR-265 on the PqsR protein. Normally, a negative binding affinity indicates the possibility of small molecules binding to proteins, while a value less than −6 kcal/mol indicates a higher binding possibility. The result showed a binding affinity score of −7.5 kcal/mol for bakuchiol to the PqsR protein, indicating a high binding affinity between bakuchiol and the PqsR protein. This is consistent with the above results.

**Figure 5:**
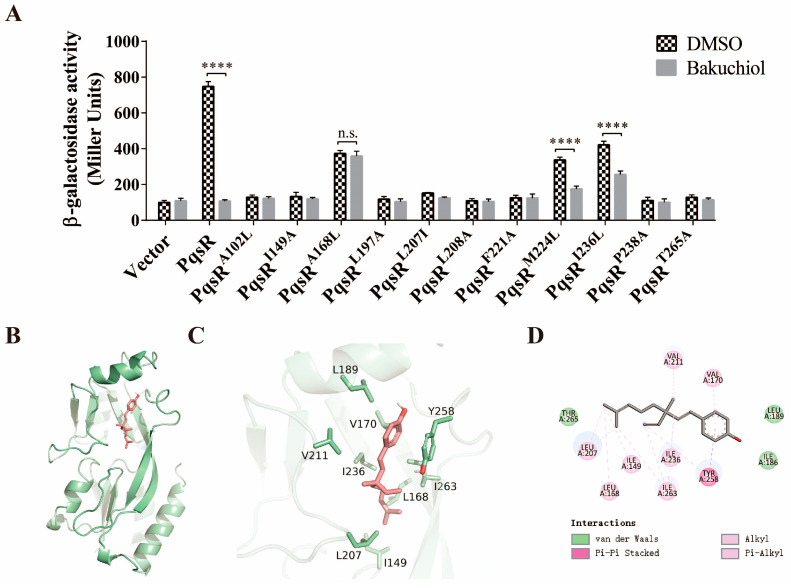
Potential target analysis of bakuchiol on PqsR. (**A**) Levels of *pqsA* transcription after site-specific mutation of PqsR (with or without bakuchiol addition). (**B**) Surface mode diagram of bakuchiol and PqsR^A168L^. (**C**) Three-dimensional interaction diagram. (**D**) Two-dimensional interaction diagram. Binding energy is −5.64 kcal/mol. All data represent results of at least three independent biological replicates. Error bars represent standard deviations. Student’s *t* test; ****: *p* < 0.0001; n.s.: *p* > 0.05.

To further verify the interaction between bakuchiol and PqsR, we constructed site-directed mutagenesis complementary vectors for these amino acid sites (MET-224, LEU-207, LEU-197, ALA-102, ALA-168, ILE-149, PRO-238, ILE-236, PHE-221, LEU-208, and THR-265). Then, these vectors were transformed into Δ*pqsR*::pMini-CTX-*pqsA’*-*lacZ*, and the expression of the *pqsA* gene was detected, as shown in Figure 5A. The results showed that compared with PqsR, PqsR^A102L^, PqsR^I149A^, PqsR^L197A^, PqsR^L207I^, PqsR^L208A^, PqsR^F221A^, PqsRP^238A^, and PqsR^T265A^ almost eliminated the expression of *pqsA* in Δ*pqsR*::pMini-CTX-*pqsA’*-*lacZ*, indicating that these sites are key active sites related to PqsR transcription. Although these site mutations did not respond to the inhibitory effect of bakuchiol, their mutations practically inactivated PqsR, making it difficult to determine if they are the sites of action of bakuchiol. In addition, compared with complementary PqsR, although mutations in ALA-168, MET-224, and ILE-236 weakened the expression of *pqsA*, PqsR^A168L^, PqsR^M224L^, and PqsR^I236L^ still regulated the transcriptional activation of *pqsA*. After adding bakuchiol, the expression activity of *pqsA* in PqsR^M224L^and PqsR^I236L^ was further reduced, which is consistent with the results of complementary PqsR, indicating that MET-224 and ILE-236 are not targets of bakuchiol. However, unlike complementary PqsR, bakuchiol did not inhibit the expression of *pqsA* after complementary PqsR^A168L^. Molecular docking analysis results showed that the binding affinity score of bakuchiol to PqsR^A168L^ was −5.64 kcal/mol (Figure 5B–D). Compared with the wild-type protein PqsR (−7.5 kcal/mol), PqsR^A168L^ had a greatly reduced binding ability to bakuchiol. These results indicated that ALA-168 is a key site for the interaction between bakuchiol and PqsR.

### 2.5. Bakuchiol Inhibits the Expression of Virulence Factors in P. aeruginosa

#### 2.5.1. Bakuchiol Inhibits the Production of Pyocyanin in *P. aeruginosa*

**Figure 6:**
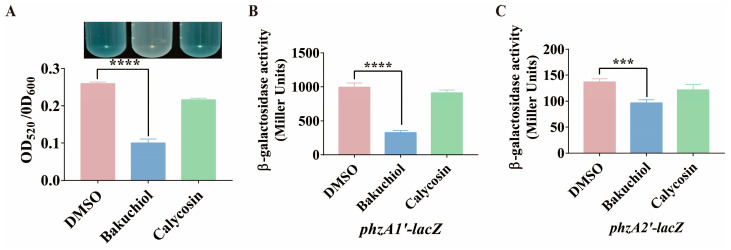
The effect of bakuchiol on the synthesis of pyocyanin by *P. aeruginosa*. (**A**) Quantitative estimation of pyocyanin. (**B**,**C**) Effects of 80 μg/mL of bakuchiol on the expression of pyocyanin synthesis genes (*phzA1* and *phzA2*). DMSO and calycosin were used as the solvent negative control and Chinese medicine monomer negative control, respectively. All data represent the results of at least three independent biological replicates. The error bars represent the standard deviations. Student’s *t* test; ***: *p* < 0.001; ****: *p* < 0.0001.

Pyocyanin plays an important role in *P. aeruginosa* infection, and its synthesis is regulated by the *pqs* system [38,39]. Two homologous seven-gene operons are responsible for the synthesis of pyocyanin, namely *phzA1B1C1D1E1F1G1* (*phzA1*) and *phzA2B2C2D2E2F2G2* (*phzA2*) [40,41]. DMSO and calycosin were used as the solvent negative control and Chinese medicine monomer negative control, respectively. We examined the effect of 80 μg/mL of bakuchiol on the production of pyocyanin by *P. aeruginosa* PAO1. The results showed that compared with the control groups, the production of pyocyanin in the treatment group was significantly reduced (Figure 6A). In addition, we constructed chromosomal *lacZ* transcriptional fusion reporter strains (PAO1::pMini-CTX-*phzA1’*-*lacZ* and PAO1::pMini-CTX-*phzA2’*-*lacZ*). Then, we detected the expression of the pyocyanin synthesis gene operons *phzA1* and *phzA2*. The results showed that compared with the control groups, the expression levels of *phzA1* and *phzA2* in the treatment group were significantly reduced (Figure 6B,C). These results indicated that 80 μg/mL of bakuchiol significantly inhibits the production of pyocyanin by *P. aeruginosa* via suppressing the *pqs* system.

#### 2.5.2. Bakuchiol Reduces the Expression of Hydrogen Cyanide, Elastase, and Lectin Synthesis Genes in *P. aeruginosa*

**Figure 7:**
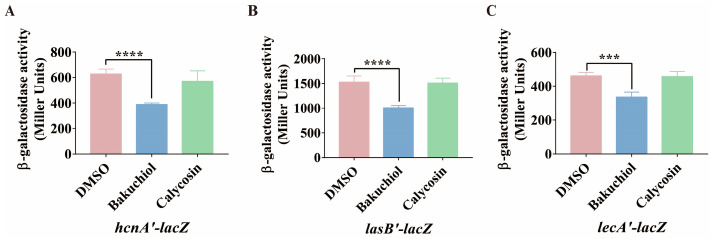
The effect of bakuchiol on the expression of various virulence factor genes in *P. aeruginosa*. (**A**–**C**) The effect of bakuchiol on (**A**) hydrogen cyanide, (**B**) elastase, and (**C**) lectin synthesis genes in *P. aeruginosa*. DMSO and calycosin were used as the solvent negative control and Chinese medicine monomer negative control, respectively. All data represent the results of at least three independent biological replicates. The error bars represent the standard deviations. Student’s *t* test; ***: *p* < 0.001; ****: *p* < 0.0001.

In addition to pyocyanin, this study also examined the effects of bakuchiol on the expression of genes involved in the synthesis of hydrogen cyanide (*hcnA*), elastase (*lasB*), and lectin (*lecA*) in *P. aeruginosa*. DMSO and calycosin were used as the solvent negative controls and Chinese medicine monomer negative control, respectively. We constructed chromosomal *lacZ* transcriptional fusion reporter strains (PAO1::pMini-CTX-*hcnA’*-*lacZ*, PAO1::pMini-CTX-*lasB’*-*lacZ*, and PAO1::pMini-CTX-*lecA’*-*lacZ*) and detected the β-galactosidase activity of these reporter strains. The results showed that compared with the control groups, the addition of 80 μg/mL of bakuchiol significantly reduced the expression levels of *hcnA*, *lasB*, and *lecA* in *P. aeruginosa* (Figure 7). These results indicated that 80 μg/mL of bakuchiol can significantly inhibit the synthesis of hydrogen cyanide, elastase, and lectin by suppressing the *pqs* system.

#### 2.5.3. Bakuchiol Reduces the Motility of *P. aeruginosa*

**Figure 8:**
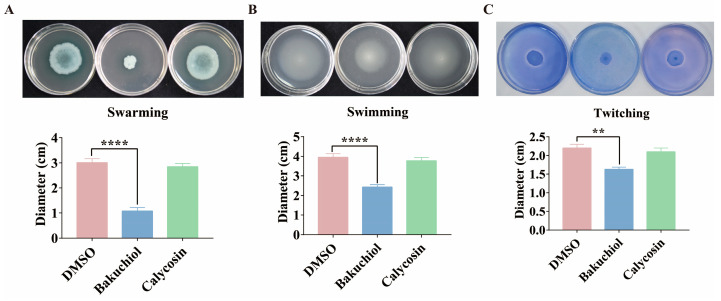
The effect of bakuchiol on the motility of *P. aeruginosa*. The effect of bakuchiol on the (**A**) swarming, (**B**) swimming, (**C**) and twitching motility in *P. aeruginosa*. DMSO and calycosin were used as the solvent negative control and Chinese medicine monomer negative control, respectively. All data represent the results of at least three independent biological replicates. The error bars represent the standard deviations. Student’s *t* test; **: *p* < 0.01; ****: *p* < 0.0001.

*P. aeruginosa* has three main modes of movement, namely swimming motility, swarming motility, and twitching motility [42,43,44]. Previous studies have reported that the *pqs* system of *P. aeruginosa* positively regulates swarming and twitching motility [45,46]. Swimming has been reported to be associated with the Type IV pili, polar fimbriae, and flagella of *P. aeruginosa* [47], but whether swimming motility in *P. aeruginosa* is affected by PQS is still unknown. This study further investigated the effect of bakuchiol on the motility of *P. aeruginosa*. The results showed that compared with the DMSO and calycosin treatment, the migration distance of *P. aeruginosa* on the swarming, swimming, and twitching plates was reduced after treatment with 80 μg/mL of bakuchiol, indicating that the addition of bakuchiol significantly inhibits the swarming, swimming, and twitching motility of *P. aeruginosa* (Figure 8).

#### 2.5.4. Bakuchiol Reduces the Pathogenicity of *P. aeruginosa*

**Figure 9:**
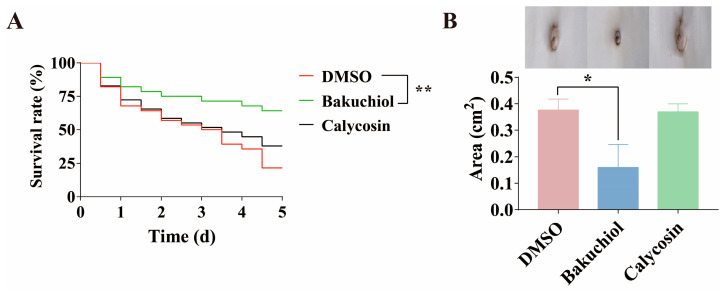
The effect of bakuchiol on the pathogenicity of *P. aeruginosa*. (**A**,**B**) Determination of the (**A**) survival rate of *Caenorhabditis elegans* and the (**B**) decay area of Chinese cabbage (**B**) in an infection model of *P. aeruginosa*. All data represent the results of at least three independent biological replicates. The error bars represent the standard deviations. Student’s *t* test; *: *p* < 0.05; **: *p* < 0.01.

PQS-mediated virulence was previously reported to endanger the survival of *Caenorhabditis elegans* [48]. As such, this study used a *C. elegans* infection model to evaluate the effect of bakuchiol on the virulence of *P. aeruginosa*. The results showed that compared with the DMSO and calycosin treatment, bakuchiol significantly increased the survival rate of *C. elegans* in this infection model (Figure 9A), indicating that bakuchiol can reduce the pathogenicity of *P. aeruginosa* in *C. elegans*.

In addition to animals, *P. aeruginosa* has also been found to be able to infect plants [49,50,51,52]. Previous studies reported a method for evaluating the infection ability of *P. aeruginosa* using Chinese cabbage [53]. Thus, we employed this plant infection model to evaluate the effect of bakuchiol on the virulence of *P. aeruginosa*. The results showed that compared with the DMSO and calycosin treatment, the decay area caused by *P. aeruginosa* infection in Chinese cabbage was significantly reduced after treatment with bakuchiol (Figure 9B). This result indicated that bakuchiol can reduce the pathogenicity of *P. aeruginosa* in Chinese cabbage.

## 3. Discussion

*P. aeruginosa* is an opportunistic pathogen that can easily cause infections in immunocompromised patients and is a common pathogen in hospitals [54]. With the overuse of antibiotics, the antibiotic resistance of *P. aeruginosa* is increasing and as a result, the development of new antibacterial strategies is imperative [55]. Drugs that suppress bacterial virulence rather than kill bacteria are important strategies currently used to address *P. aeruginosa* resistance [56]. QS systems are crucial for *P. aeruginosa* to regulate various life activities and synthesize numerous virulence factors [57]. Therefore, QS systems can become potential drug targets. Traditional Chinese medicine has a long history in medical treatment in China, and its active ingredients have been widely developed into antibacterial agents [58,59,60]. At present, various traditional Chinese medicine monomers have been reported to have anti-QS activity, including vanillin, gingerol, and baicalin [35,61,62]. To screen for active substances with anti-*P. aeruginosa* QS system activity, we established a traditional Chinese medicine monomer library and used a *lacZ*-based transcriptional fusion biosensor for screening anti-QS activities. The screening results showed that bakuchiol possesses significant anti-QS activity.

Previous studies have shown that bakuchiol can inhibit the *las* system of *P. aeruginosa* [34]. Under normal circumstances, the LuxR receptor, LasR, functions in a dimeric form [34]. The presence of bakuchiol can interfere with the normal function of LasR by disrupting its dimerization [34]. Surprisingly, contrary to reports of bakuchiol inhibiting the *las* system, our results showed that bakuchiol did not exert an inhibitory effect on the *las* system but rather inhibited the function of the *pqs* system in a concentration-dependent manner (Figure 1 and Figure 2). The reason for this differential phenotype may be the result of different experimental conditions. A previous study used a strategy of detecting reporter genes in a heterologous environment (*E. coli*, not *P. aeruginosa*) [34]. Although, as they stated, this method avoids interference from other *P. aeruginosa* QS systems, different physiological environments may affect protein function and activity. Furthermore, the concentration of bakuchiol was lower than the concentration used in this study [34]. Our strategy was to conduct in situ experiments in *P. aeruginosa*, which may require a higher concentration of bakuchiol for the *P. aeruginosa* system. In addition, according to the literature reports, the minimum odDHL (3-oxo-C_12_-HSL) concentration required for the LasR activity of *P. aeruginosa* is about 20 nM [63], which is much higher than the odDHL concentration (2 nM) added by the Alasiri et al. in the *E. coli* system [34]. The difference in concentration may result in different outcomes for the two systems. By contrast, the in situ experimental strategy adopted in this study is more conducive to exploring the effect of bakuchiol on *P. aeruginosa*. In the *las* system, LasR dimerizes after binding to odDHL and then activates the expression of downstream genes [14]. However, in the work of Alasiri et al. [34], only the interaction analysis of odDHL and bakuchiol with LasR was carried out in the thermal shift assay and sedimentation velocity analysis, and the competitive interference test of the combination of odDHL and LasR by bakuchiol was not performed. In addition, LasR without a ligand was not used as a control in these experiments. Therefore, the conclusion that bakuchiol targets LasR is not reliable.

To further confirm that bakuchiol targets the *pqs* system and not the *las* system, we conducted investigations into its inhibitory mechanism. Traditional Chinese medicine monomers can interact with target proteins, alter their conformation, and cause changes in their function [64]. According to reports, traditional Chinese medicine monomer vanillin may inhibit the *pqs* system by binding to PqsR [35]. Similarly, through genetic complementation analysis, we found that bakuchiol lost its ability to inhibit *pqsA* gene expression when the *pqsR* gene was overexpressed (Figure 3). Furthermore, molecular dynamics simulations have shown that bakuchiol interacts with multiple amino acid residues on the PqsR protein through hydrophobic or hydrogen-bonding interactions (Figure 4), indicating that bakuchiol may alter the conformation and function of PqsR. Based on this, we analyzed the key active sites involved in the binding of bakuchiol to PqsR. The results showed that the amino acid residue ALA-168 plays a crucial role in both the binding of bakuchiol to PqsR and the inhibition of its function (Figure 5). After mutating other amino acid sites, such as ALA-102, ILE-149, LEU-197, LEU-207, LEU-208, PHE-221, PRO-238, and THR-265, the activity of PqsR almost disappeared. A previous study also demonstrated that the mutation of ILE-149, LEU-207, and PHE-221 results in an almost complete loss of the transcriptional activity of PqsR [65]. Compared with a previous report, this study found that ALA-102, LEU-197, LEU-208, PRO-238, and THR-265 are also key active sites of PqsR. Because the mutations in ALA-102, ILE-149, LEU-197, LEU-207, LEU-208, PHE-221, PRO-238, and THR-265 resulted in an almost complete loss of PqsR activity, it was difficult to determine whether they are the sites of action for bakuchiol. To demonstrate that bakuchiol can directly bind to PqsR, we also attempted to purify the PqsR protein for an electrophoretic mobility shift assay and isothermal titration calorimetry experiments. Unfortunately, we were unable to successfully purify the full-length PqsR protein. This is consistent with previous studies, where a full-length PqsR protein was unable to be purified and researchers instead used the co-inducer-binding domain (CBD) of PqsR for analysis [65].

In addition to exploring the molecular mechanism of bakuchiol in inhibiting the *pqs* system of *P. aeruginosa*, this study also evaluated the effect of bakuchiol on the pathogenicity of *P. aeruginosa*. The pathogenicity of *P. aeruginosa* depends on virulence factors and motility [11,66]. Therefore, inhibiting the production of virulence factors and motility is also considered an important means of treating *P. aeruginosa* infection [66]. Pyocyanin is one of the most important virulence factors produced by *P. aeruginosa* and plays a crucial role in infection [67]. Its synthesis is regulated by QS systems and is commonly used as a biomarker for evaluating QS behavior [67]. According to our results, bakuchiol reduced the production of *P. aeruginosa* pyocyanin (Figure 6A). This is because bakuchiol reduced the expression levels of pyocyanin synthesis genes *phzA1* and *phzA2* in *P. aeruginosa* (Figure 6B,C). This is consistent with the results of Alasiri et al., indicating that bakuchiol has an inhibitory effect on QS systems. In addition, we found that bakuchiol significantly reduced the expression of other virulence factor synthesis genes regulated by the *pqs* system in *P. aeruginosa*, including hydrogen cyanide, elastase, and lectin synthesis genes (*hcnA*, *lasB*, and *lecA*) (Figure 7). Previous studies have shown that bakuchiol reduces the formation of biofilms in *P. aeruginosa* [34]. Generally speaking, *P. aeruginosa* must adhere to biological or non-biological surfaces to form biofilms, which is accompanied by a decrease in motility. Although our results indicated that bakuchiol did not significantly affect biofilm formation, we found that all three motility types of *P. aeruginosa* were significantly inhibited by bakuchiol (Figure 8). These results indicated that bakuchiol reduces the production of virulence factors and motility, suggesting that the pathogenicity of *P. aeruginosa* may also have changed. We found that bakuchiol can exert a certain protective function on both Chinese cabbage and *C. elegans* (Figure 9) against *P. aeruginosa*. Bakuchiol was able to not only protect Chinese cabbage from *P. aeruginosa* infection but also reduce *P. aeruginosa* pathogenicity in *C. elegans*.

## 4. Materials and Methods

### 4.1. Monomers Used in Traditional Chinese Medicine

Traditional Chinese medicine monomers were purchased from Baoji Chengguang Biotechnology Co., Ltd. (Baoji, China), with a purity of over 98%. The CAS number for bakuchiol was 10309-37-2, and the CAS number for calycosin was 20575-57-9. Both bakuchiol and calycosin were prepared in DMSO at a concentration of 50 mg/mL. Both solutions were stored at −20 °C until future use.

### 4.2. Bacterial Strains, Plasmids, Primers, and Culture Conditions

The bacterial strains and plasmids used in the study are listed in Table S3. The primers are listed in Table S4. Both *P. aeruginosa* and *E. coli* were cultured statically at 37 °C or cultured at 37 °C with shaking at 200 rpm. Luria–Bertani (LB) medium was used to cultivate *P. aeruginosa* and *E. coli*, while *Pseudomonas* broth medium [68] was used to measure the production of pyocyanin by *P. aeruginosa*. Swarming medium, swimming medium, and twitching medium were used to detect the motility of *P. aeruginosa* [53,69]. Nematode growth medium (NGM) was used to cultivate *C. elegans* [53]. According to the specific experimental requirements, the culture medium was supplemented with kanamycin (Km, 30 μg/mL), tetracycline (Tc, 200 μg/mL), and gentamicin (Gm, 100 μg/mL). The formulas of culture medium used in this study are shown in Table S5.

### 4.3. Plasmid Construction

Plasmids were modified and constructed based on previously reported studies [1,70]. To construct the complementary plasmid pBBR1MCS-5-*pqsR*, the PCR-amplified *pqsR gene* containing the native Shine–Dalgarno (SD) sequence was cloned into the *Xho* I and *Pst* I sites of the plasmid pBBR1MCS-5. This resulted in the recombinant plasmid pBBR1MCS-5-*pqsR*. Site-directed mutagenesis was modified and conducted based on previously reported studies [2,71].

### 4.4. Construction of In-Frame Deletion Mutant

Construction of the knock-out plasmid was modified from previously reported studies [1,70]. Briefly, to construct the recombinant suicide plasmids for deletion, 1106 bp upstream and 1173 bp downstream of the *pqsR* gene were amplified using the primer pairs *pqsR* up F/*pqsR* up R and *pqsR* low F/*pqsR* low R, respectively (Supplementary Table S4). The upstream and downstream PCR fragments were ligated using overlapping PCR, and the resulting PCR products were inserted into the EcoR I/Hind III sites of the suicide vector pK18*mobsacB* to yield the plasmid p-*pqsR*. The GM resistance cassette from p34s-Gm was subsequently inserted into the same Hind III site of p-*pqsR* to yield the recombinant suicide plasmid pK-Δ*pqsR*-Gm. pK18-Δ*pqsR*-Gm was transformed into *E. coli* S17-1 and then conjugated with *P. aeruginosa* PAO1 to obtain the single cross-over mutants. The Δ*pqsR* mutant strain was obtained through sucrose lethal screening and resistance screening.

### 4.5. Construction of lacZ Chromosomal Fusion Reporter Strains

The *lacZ* chromosomal fusion reporter strains were constructed and modified based on previously reported studies [1,70]. Taking the *pqsA* gene as an example, the promoter transcription fusion vector pMini-CTX-*pqsA’*::*lacZ*, a 1036 bp promoter DNA sequence of the *pqsA* gene (PpqsA), was amplified using primers *pqsA* F/R. PCR-amplified PpqsA was cloned into the *Sal* I and *Pst* I sites of the plasmid pMini-CTX::*lacZ*, producing recombinant plasmid pMini-CTX-*pqsA’*::*lacZ*. The obtained recombinant vector pMini-CTX-*pqsA’*::*lacZ* was transformed into *E. coli* S17-1. Subsequently, the recombinant vector was integrated into the CTX phage attachment site (*attB*) of PAO1 using the conjugation method. Finally, chromosome transcription fusion reporter strains were obtained through resistance screening (Km, 30 μg/mL and Tc, 200 μg/mL). Using the same method, this study obtained other gene promoter transcription fusion reporter strains.

### 4.6. Site-Directed Mutagenesis

To construct the PqsR^A168L^ point mutant, the alanine residues at position 168 of PqsR were replaced by leucine residues. The front and rear sections of the DNA sequences of the *pqsR* gene were amplified using the primer pairs pUC18T upF/168 upR and 168 lowF/pUC18T lowR, respectively, and the upstream and downstream sequences were connected using overlapping PCR to produce pqsR^A168L^ containing its own promoter. The product of overlapping PCR was inserted into the *Hind* III/*Sac* I sites of the pUC18T-mini-Tn7T-Gm plasmid to yield the site-directed mutagenesis recombinant plasmid pUC18T-mini-Tn7T-*pqsR*^A168L^-Gm. Assisted by the pTNS3 plasmid, pUC18T-mini-Tn7T-*pqsR*^A168L^-Gm was transformed into Δ*pqsR*::pMini-*pqsA’*-*lacZ* to obtain ALA-168 site-directed mutant Δ*pqsR* (pUC18T-mini-*pqsR*^A168L^-Tn7T-Gm)::pMini-*pqsA’*-*lacZ*. Point mutation mutants (Table S3) at other sites were obtained by the same method.

### 4.7. β-Galactosidase Activity Assays

The β-galactosidase activity assays were modified and conducted based on previously reported studies [1,70]. First, 420 µL Z buffer (60 mmol/L Na_2_HPO_4_, 40 mmol/L NaH_2_PO_4_, 10 mmol/L KCl, 1 mmol/L MgSO_4_, pH = 7.0, 0.2% β-mercaptoethanol), 20 µL chloroform, and 10 µL 0.1% sodium dodecylsulfate (SDS) were added to 50 µL logarithmic phase bacterial suspension. After rapid mixing of the suspension for 20 s, the sample was incubated at 30 °C for 1 h, and then 100 µL of 4 mg/mL 2-nitrophenyl β-d-galactopyranoside (ONPG, Sigma, St. Louis, MO, USA) was added to the mixture. The reaction was terminated by adding 250 µL 1 mmol/L Na_2_CO_3_, and the reaction time was recorded. The mixture was centrifuged at 14,000× *g* for 3 min, and then the optical density of the supernatant was measured at 420 and 550 nm. Finally, the activity of β-galactosidase in Miller units (MUs) was calculated according to the following equation:

$$\mathrm{MU}=\frac{1000\times \left({\mathrm{OD}}_{420}-1.75\times {\mathrm{OD}}_{550}\right)}{\mathrm{Time}\;\left(\min\right)\times \mathrm{Volume}\;\left(\mathrm{mL}\right)\times {\mathrm{OD}}_{600}}$$

### 4.8. Growth Assays

*P. aeruginosa* PAO1 was cultured overnight in an LB liquid medium. Then, the culture was subcultured into fresh LB liquid medium (containing DMSO, bakuchiol, or calycosin) at 1:100. Finally, the culture was incubated under shaking conditions at 37 °C at 220 rpm. The optical densities of the culture were read at 600 nm, and the growth curve was plotted.

### 4.9. Measurement of Pyocyanin

The measurement of pyocyanin was modified and conducted based on a previously reported study [68]. Specifically, *P. aeruginosa* PAO1 was cultured overnight in an LB liquid medium. The culture was then subcultured in *Pseudomonas* broth medium at 1:100 and incubated at 37 °C, at 200 rpm until the stationary phase. The cultures were then centrifuged at 12,000 rpm for 2 min, and the supernatant was collected. Then, chloroform was added to the supernatant, which was then mixed for 2 min and then centrifuged at 8000 rpm for 10 min. The chloroform layer was collected, and then 0.2 mol/L HCl was added to the chloroform layer and mixed. Finally, the samples were centrifuged at 8000 rpm for 10 min. The optical densities of the pink extract were read at 520 nm. The production of pyocyanin (μg/mL) was then calculated according to the following equation:

$$\mathrm{pyocyanin}\;\left(\mu g/\mathrm{mL}\right)=\frac{{\mathrm{OD}}_{520}}{{\mathrm{OD}}_{600}}\times 17.072$$

### 4.10. Motility Assays

Motility assays were modified and conducted based on previously reported studies [53,69]. DMSO, bakuchiol, or calycosin was added to the culture media during the experiment. All plates were dried of surface moisture under sterile conditions before use. PAO1 culture (2 μL) was inoculated at the center of the surface of the swarming and swimming plates and cultured at 37 °C for 24 h. The diameter of each motility zone was measured. The twitching motility was observed by stab-inoculating PAO1 through the twitching plate, which was inverted and cultured at 37 °C for 24 h. Then, the twitching PAO1 cells were stained with Coomassie brilliant blue staining solution (0.5 g/L Coomassie Brilliant Blue R250, 400 mL/L methanol, 100 mL/L acetic acid) and decolored with anhydrous ethanol to determine diameters.

### 4.11. Chinese Cabbage Infection Assay

The Chinese cabbage infection assay was modified and conducted based on previously reported studies [72,73]. PAO1 was grown overnight in an LB liquid medium, and then the cultures were harvested. The cells were washed with 10 mmol/L MgSO_4_ twice to remove nutrient-rich substances and then diluted to 10^8^ CFU/mL. After the surface of the Chinese cabbage was disinfected with 0.1% H_2_O_2_, 10 µL of the diluted bacterial suspension (containing DMSO, bakuchiol, or calycosin) was injected with a syringe into the midrib of the Chinese cabbage leaves. The specimens were kept at 30 °C for three days, and rot symptoms were monitored throughout this period.

### 4.12. Anti-Infective Activity Assay of C. elegans

An anti-infective activity assay of *C. elegans* was modified and conducted based on a previously reported study [53]. PAO1 suspension was cultured to OD_600_ = 2.0 and evenly spread on NGM plates (diameter of 3.5 cm). The plates were cultivated at 37 °C for 12 h and then cultivated at 25 °C for another 12 h. *C. elegans* was synchronized to the L4 phase and then transferred to the NGM plates. The survival rate of *C. elegans* was recorded every 12 h for 5 d.

### 4.13. Molecular Dynamics Simulation

The molecular dynamics simulation was modified and conducted based on previously reported studies [74,75,76]. In short, bakuchiol was used to dock the transcriptional activator protein PqsR of the *P. aeruginosa* PAO1 *pqs* system. The 3D crystal structure of PqsR (PDB ID: 4JVD) was downloaded from the Protein Data Bank (http://www.pdb.org). The 3D structure of bakuchiol was constructed using Chem3D 14.1, and energy minimization was performed under an MMFF94 force field. The 3D crystal structure of bakuchiol was docked to the binding sites of the PqsR protein using AutoDock Vina 1.1.2 [74,75]. Finally, the molecular docking results were visualized and analyzed using PyMol 2.5.4 and Discovery Studio [76].

### 4.14. Statistical Analysis

All experiments were conducted in triplicate and repeated on two different occasions. The data are expressed as mean ± SD (standard deviation). Student’s *t* test (bilateral and unpaired) was performed using GraphPad Prism version 7.00 software (GraphPad Software Inc., San Diego, CA, USA). A *p*-value < 0.05 was considered statistically significant. Unless otherwise specified, all figures were created using ChemDraw (PerkinElmer, Waltham, CA, USA), GraphPad Prism 7.00, and Adobe Illustrator 2020 (CS6; Adobe, Mountain View, CA, USA).

## 5. Conclusions

The results of this study demonstrate that bakuchiol inhibits the *pqs* system of *P. aeruginosa* by targeting the transcriptional regulatory protein PqsR, thereby reducing the production of virulence factors and its motility. In addition, bakuchiol reduced the pathogenicity of *P. aeruginosa* to Chinese cabbage and *C. elegans*. In summary, bakuchiol is an effective *pqs* system inhibitor that can alleviate infections caused by *P. aeruginosa*.

## Data Availability

The original contributions presented in this study are included in the article and Supplementary Materials. Further inquiries can be directed to the corresponding authors.

## Supplementary Materials

The following supporting information can be downloaded at: https://www.mdpi.com/article/10.3390/ijms26010243/s1. References [53,68,69,71,77,78,79,80,81,82] are cited in the Supplementary Materials.
