# Supplementary material for: Traditional Chinese Medicine Monomer Bakuchiol Attenuates the Pathogenicity of Pseudomonas aeruginosa via Targeting PqsR
Source: Int J Mol Sci. 2024 Dec 30;26(1):243. doi: 10.3390/ijms26010243 (PMC11719591; doi:10.3390/ijms26010243)
Supplement: Supplementary file 1 [file ijms-26-00243-s001.zip › ijms-3391615-supplementary.pdf]

Supplemental Materials

Table S1. Screening of traditional Chinese medicine monomers that inhibit the expression of *pqsA*, *lasI*, and *rhlI* in *P. aeruginosa*

Number	Traditional Chinese medicine monomers	CAS	Inhibition effect on QS		
			pqsA	*lasI*	*rhlI*
1	Eupatilin	22368-21-4	-	-	-
2	Abietic acid	514-10-3	-	-	-
3	Orientin	28608-75-5	-	-	-
4	Bakuchiol	10309-37-2	+ + +	-	-
5	Ingeno	30220-46-3	-	-	-
6	Aucubin	479-98-1	-	-	-
7	P-Hydroxybenzoic acid	99-96-7	-	-	-
8	Calycosin	20575-57-9	-	-	-
9	wogonoside	51059-44-0	-	-	-
10	Madecassic acid	34540-22-2	+ +	-	-
11	Triptophenolide	74285-86-2	-	-	-
12	Wilforlide A	84104-71-2	-	-	-
13	Forsythin	487-41-2	-	-	-
14	Orcinol glucoside	21082-33-7	-	-	-
15	Artemisinic acid	80286-58-4	-	-	-
16	Casticin	479-91-4	-	-	-
17	Parishin A	62499-28-9	-	-	-
18	Saikosaponin D	20874-52-6	-	-	-
19	Deoxyschizandrin	61281-38-7	-	-	-
20	Aloe-emodin	481-72-1	-	-	-

+++: strong inhibitory activity; ++: moderate inhibitory activity; –: no inhibitory activity.

Table S2. MIC analysis of bakuchiol on *P. aeruginosa*

Compound	Concentration μg/mL								
Bakuchiol	0	1	2	4	8	16	32	64	128
Results	+	+	+	+	+	+	+	+	+

Note: "+" represents normal bacterial growth; bakuchiol precipitates (the concentration>128 μg/mL)

Table S3. List of strains and plasmids used in this study

Strains and plasmids	Relevant characteristics	Source
Strains		
P. aeruginosa		
PAO1 (ATCC15692)	Wild-type	This lab
Δ*pqsR*	Mutant of knockout *pqsR* in PAO1	This study
PAO1 (pBBR1MCS-5)	PAO1 containing pBBR1MCS-5	This study
PAO1 (pBBR1MCS-5-*pqsR*)	PAO1 containing pBBR1MCS-5-*pqsR*	This study
PAO1 (pBBR1MCS-5)::pMini-CTX-*pqsA′-lacZ*	PAO1 containing pMini-CTX-*pqsA′*::*lacZ* with pBBR1MCS-5	This study
PAO1 (pBBR1MCS-5-*pqsR*)::pMini-CTX-*pqsA′-lacZ*	PAO1 containing pMini-CTX-*pqsA′*::*lacZ* with pBBR1MCS-5-*pqsR*	This study
PAO1::pMini-CTX-*pqsA′-lacZ*	PAO1 containing pMini-CTX-*pqsA′*::*lacZ*	This study
PAO1::pMini-CTX-*rhlI′-lacZ*	PAO1 containing pMini-CTX- *rhlI′*::*lacZ*	This study
PAO1::pMini-CTX-*lasI′-lacZ*	PAO1 containing pMini-CTX- *lasI′*::*lacZ*	This study
PAO1::pMini-CTX-*phzA1′-lacZ*	PAO1 containing pMini-CTX-*phzA1′*::*lacZ*	This study
PAO1::pMini-CTX-*phzA2′-lacZ*	PAO1 containing pMini-CTX-*phzA2′*::*lacZ*	This study
PAO1::pMini-CTX-*lecA′-lacZ*	PAO1 containing pMini-CTX-*lecA′*::*lacZ*	This study
PAO1::pMini-CTX-*hcnA′-lacZ*	PAO1 containing pMini-CTX-*hcnA′*::*lacZ*	This study
PAO1::pMini-CTX-*lasB′-lacZ*	PAO1 containing pMini-CTX-*lasB′*::*lacZ*	This study
Δ*pqsR*::pMini-*pqsA′-lacZ*	Δ*pqsR* containing pMini-CTX-*pqsA′*::*lacZ*	This study
Δ*pqsR* (pUC18T-mini-*pqsR*^{A102L}-Tn7T-Gm)::pMini-*pqsA′-lacZ*	Δ*pqsR* containing pMini-*pqsA′*::*lacZ* with pUC18T-mini-*pqsR*^{A102L}-Tn7T-Gm	This study
Δ*pqsR* (pUC18T-mini-*pqsR*^{I149A}-Tn7T-Gm)::pMini-*pqsA′-lacZ*	Δ*pqsR* containing pMini-*pqsA′*::*lacZ* with pUC18T-mini-*pqsR*^{I149A}-Tn7T-Gm	This study
Δ*pqsR* (pUC18T-mini-*pqsR*^{A168L}-Tn7T-Gm)::pMini-*pqsA′-lacZ*	Δ*pqsR* containing pMini-*pqsA′*::*lacZ* with pUC18T-mini-*pqsR*^{A168L}-Tn7T-Gm	This study
Δ*pqsR* (pUC18T-mini-*pqsR*^{L197A}-Tn7T-Gm)::pMini-*pqsA′-lacZ*	Δ*pqsR* containing pMini-*pqsA′*::*lacZ* with pUC18T-mini-*pqsR*^{L197A}-Tn7T-Gm	This study
Δ*pqsR* (pUC18T-mini-*pqsR*^{L207I}-Tn7T-Gm)::pMini-*pqsA′-lacZ*	Δ*pqsR* containing pMini-*pqsA′*::*lacZ* with pUC18T-mini-*pqsR*^{L207I}-Tn7T-Gm	This study
Δ*pqsR* (pUC18T-mini-*pqsR*^{L208A}-Tn7T-Gm)::pMini-*pqsA′-lacZ*	Δ*pqsR* containing pMini-*pqsA′*::*lacZ* with pUC18T-mini-*pqsR*^{L208A}-Tn7T-Gm	This study
Δ*pqsR* (pUC18T-mini-*pqsR*^{F221A}-Tn7T-Gm)::pMini-*pqsA′-lacZ*	Δ*pqsR* containing pMini-*pqsA′*::*lacZ* with pUC18T-mini-*pqsR*^{F221A}-Tn7T-Gm	This study
Δ*pqsR* (pUC18T-mini-*pqsR*^{M224L}-Tn7T-Gm)::pMini-*pqsA′-lacZ*	Δ*pqsR* containing pMini-*pqsA′*::*lacZ* with pUC18T-mini-*pqsR*^{M224L}-Tn7T-Gm	This study
Δ*pqsR* (pUC18T-mini-*pqsR*^{I236L}-Tn7T-Gm)::pMini-*pqsA′-lacZ*	Δ*pqsR* containing pMini-*pqsA′*::*lacZ* with pUC18T-mini-*pqsR*^{I236L}-Tn7T-Gm	This study
Δ*pqsR* (pUC18T-mini-*pqsR*^{P238A}-Tn7T-Gm)::pMini-*pqsA′-lacZ*	Δ*pqsR* containing pMini-*pqsA′*::*lacZ* with pUC18T-mini-*pqsR*^{P238A}-Tn7T-Gm	This study
Δ*pqsR* (pUC18T-mini-*pqsR*^{T265A}-Tn7T-Gm)::pMini-*pqsA′-lacZ*	Δ*pqsR* containing pMini-*pqsA′*::*lacZ* with pUC18T-mini-*pqsR*^{T265A}-Tn7T-Gm	This study
Δ*pqsR* (pUC18T-mini-*pqsR*-Tn7T-Gm)::pMini-*pqsA′-lacZ*	Δ*pqsR* containing pMini-*pqsA′*::*lacZ* with pUC18T-mini-*pqsR*-Tn7T-Gm	This study

Δ*pqsR* (pUC18T-mini-Tn7T-Gm)::pMini-*pqsA′-lacZ*	Δ*pqsR* containing pMini-*pqsA′*::*lacZ* with pUC18T-mini-Tn7T-Gm	This study
Δ*pqsR* (pUC18T-mini-*pqsR*^{A102L}-lacZ)::pMini-*pqsA′-lacZ*	Δ*pqsR* containing pMini-*pqsA′*::*lacZ* with pUC18T-mini-*pqsR*^{A102L}-Gm	This study
E. coli		
TG1	[F′ *traD36*,*proAB*,*lacI*^{q},*lacZ*Δ*M15*]*supE*thi-1 Δ(*lac-proAB*),Δ(*mcrB-hsdSM*)5,(r_{K^-} m_{K^-})	This lab
OP50	Amp^r; Wild type food for *C. elegans*	[77]
S17-1	RP4-2(Km::Tn7, Tc::Mu-1), pro-82, LAMpir, *recA1*, *endA1*, *thiE1*, *hsdR17*, *creC510*	This lab
Plasmids		
pK18*mobSac*B	Km^r; sacB-based gene replacement vector	[78]
pBBR1MCS-5	Broad-host-range vector, Gm^r	[79]
p34s-Gm	Amp^r; Gm resistant cassette carrying vector	[80]
pMini-CTX-*lacZ*	Ω-FRT-*attP*-MCS, *ori*, *int*, *oriT*, Tc^r	[81]
pTNS3	Amp^r; plasmid expressing *tnsABCD* from *P1* and P_{lac}	[82]
pK18-Δ*pqsR*-Gm	Km^r; Gm^r; Δ*pqsR*::Gm in pK18*mobSac*B	This study
pBBR1MCS-5- *pqsR*	*pqsR* cloned into pBBR1MCS-5 for complementation	This study
pMini-CTX-*pqsA′*::*lacZ*	1105 bp upstream region of *pqsA* in pMini-CTX::*lacZ*	This study
pMini-CTX-*rhlI′*::*lacZ*	604 bp upstream region of *rhlI* in pMini-CTX::*lacZ*	This study
pMini-CTX-*lasI′*::*lacZ*	569 bp upstream region of *lasI* in pMini-CTX-*lacZ*	This study
pMini-CTX-*phzA1′*::*lacZ*	530 bp upstream region of *phzA1* in pMini-CTX::*lacZ*	This study
pMini-CTX- *phzA2′*::*lacZ*	525 bp upstream region of *phzA2* in pMini-CTX::*lacZ*	This study
pMini-CTX-*lecA′*::*lacZ*	536 bp upstream region of *lecA* in pMini-CTX::*lacZ*	This study
pMini-CTX-*hcnA′*::*lacZ*	945 bp upstream region of *hcnA* in pMini-CTX::*lacZ*	This study
pMini-CTX-*lasB′*::*lacZ*	1100 bp upstream region of *lasB* in pMini-CTX::*lacZ*	This study
pUC18T-mini-Tn7T-Gm	Gm^r on mini-Tn7T; mobilizable; for gene insertion in Gms bacteria	[71]
pUC18T-mini-*pqsR*^{A102L}-Tn7T-Gm	*pqsR*^{A102L} containing its own promoter was cloned into pUC18T-mini-Tn7T-Gm	This study
pUC18T-mini-*pqsR*^{I149A}-Tn7T-Gm	*pqsR*^{I149A} containing its own promoter was cloned into pUC18T-mini-Tn7T-Gm	This study
pUC18T-mini-*pqsR*^{A168L}-Tn7T-Gm	*pqsR*^{A168L} containing its own promoter was cloned into pUC18T-mini-Tn7T-Gm	This study
pUC18T-mini-*pqsR*^{L197A}-Tn7T-Gm	*pqsR*^{L197A} containing its own promoter was cloned into pUC18T-mini-Tn7T-Gm	This study
pUC18T-mini-*pqsR*^{L207I}-Tn7T-Gm	*pqsR*^{L207I} containing its own promoter was cloned into pUC18T-mini-Tn7T-Gm	This study
pUC18T-mini-*pqsR*^{L208AL}-Tn7T-Gm	*pqsR*^{L208A} containing its own promoter was cloned into pUC18T-mini-Tn7T-Gm	This study
pUC18T-mini-*pqsR*^{F221A}-Tn7T-Gm	*pqsR*^{F221A} containing its own promoter was cloned into pUC18T-mini-Tn7T-Gm	This study
pUC18T-mini-*pqsR*^{M224L}-Tn7T-Gm	*pqsR*^{M224L} containing its own promoter was cloned into pUC18T-mini-Tn7T-Gm	This study
pUC18T-mini-*pqsR*^{I236L}-Tn7T-Gm	*pqsR*^{I236L} containing its own promoter was cloned into pUC18T-mini-Tn7T-Gm	This study

pUC18T-mini-*pqsR*^{P238A}-Tn7T-Gm	*pqsR*^{P238A} containing its own promoter was cloned into pUC18T-mini-Tn7T-Gm	This study
pUC18T-mini-*pqsR* ^{T265A} -Tn7T-Gm	*pqsR*^{T265A} containing its own promoter was cloned into pUC18T-mini-Tn7T-Gm	This study
pUC18T-mini-*pqsR* -Tn7T-Gm	*pqsR* containing its own promoter was cloned into pUC18T-mini-Tn7T-Gm	This study

*Tc^{r} , Gm^{r} , Amp^{r} and Km^{r} represent resistance to tetracycline, gentamicin, kanamycin, and ampicillin, respectively.

Table S4. List of primiers used in this study

Primiers	5′-3′ Sequence
pqsR upF	AGTCGAATTCATCTCCAGCGAATCGGAT
pqsR upR	CAATACACCTTCGATTTCCAGGTTGCTG
pqsR lowF	TGGAAATCGAAGGTGTATTGCTACTACC
pqsR lowR	ACGTAAGCTTACATGCTCAAGGTCGATC
pqsA F	CTCGGTCGACAACCCACCGGCGAAACCG
pqsA R	CTCGCTGCAGGCATTGCAGCCGGCTGAG
rhlI F	GTACCTCGAGCGACCAGCAGAACATCTCC
rhlI R	CTAGAAGCTTGACCACGTCCCAGCCCAG
lasI F	GTACCTCGAGCTCGGAAGCCAATGTGAAC
lasI R	CTAGAAGCTTCTTTGCGCTCCTTGAACAC
phzA1 F	AGCTGGTACCAAAGTTTCTCCGGCATAC
phzA1 R	AGCTAAGCTTAGTGGGAATACCGTCACG
phzA2 F	AGCTGGTACCATGGATGCCAGTCGATTC
phzA2 R	AGCTAAGCTTGGTGGGAATACCGTCACG
lecA F	CTCGAGATCTTGTGTTTCCTGGCGTTCAG
lecA R	CTCGCTGCAGCTGGGTAGGTCCGTAACTG
hcnA F	CTCGGGTACCCATGAGTCGCGAAGTACG
hcnA R	CTCGCTGCAGACGGCATTGAGCACGTTG
lasB F	CTCGGGTACCGATCGGAATCCTCGCCTC
lasB R	CTCGCTGCAGGCGGATCGCTTTCAGTTC
pqsR F	CTCGCTCGAGGATGCCTATTCATAACCTG
pqsR R	CTCGCTGCAGGGAGAACGCTCTACTCTG
102 upR	GACGGCGGGATAAGGGTGTCCAGCAGCA
102 lowF	GGACACCCTTATCCCGCCGTCGTTCTG
149 upR	CAGTTCCTCGTCTGCGGTGATGGCGATA
149 lowF	CATCACCGCAGACGAGGAACTGAAGATC
168 upR	GCGGATGGGCGACGACGAACAACTTGGTGTAG
168 lowF	GTTCGTCGTCGCCCATCCGCAGCACCCG
197 upR	GGCTGCCTGCGCTGATCTGCCGGTAATT
197 lowF	AGATCAGCGCAGGCAGCCGCTCCGGGCA
207 upR	CTGACCGGCCGCAGTATGTTCGAATGCT
207 lowF	AACATACTGCGGCCGGTCAGCGACAAGG
208 upR	CGCTGACCGGCCGTGCCAGGTTCGAATG
208 lowF	GGCACGGCCGGTCAGCGACAAGGTGCTC

221 upR	GTCCGCGTTTTCCACGAAGAGCACCTTG
221 lowF	CTTCGTGGAAAACGCGGACGACATGCTG
224 upR	CCAGACGCAGTAGGTCGTCGAAGTTTTC
224 lowF	GACGACCTACTGCGTCTGGTGGAAGCC
236 upR	GAAATAATGCGGCGCAAGGCCCCATCCGAC
236 lowF	GCCTTGCGCCGCATTATTTCGTCGAGGAAC
238 upR	GTTCCTCGACGAAATAATGTGCCGCGATGCCC
238 lowF	GCACATTATTTCGTCGAGGAACGCCTGC
265 upR	TACACCTTTGCGTCGATGCCGCCCGGTTC
265 lowF	GCATCGACGCAAAGGTGTATTGCTACTAC
pUC18T upF	GTACAAGCTTGATCTTCGCCGTCTCGCCCA
pUC18T lowR	GTACGAGCTCGAACGCTCTACTCTGGTG

*Underlined sites indicate restriction enzyme cutting sites added for cloning.

Table S5. Name and formula of culture medium

Name	Formula	Source#
Luria–Bertani (LB) medium	NaCl 10 g/L, yeast extract 5 g/L, tryptone 10 g/L, agar 15 g/L, pH=7.0	-
Pseudomonas broth medium	NaCl 1.4 g/L, K_2SO_4 10 g/L, Tryptone 20 g/L	[68]
Swarming medium	nutrient broth 8 g/L, glucose 5 g/L and agarose 6 g/L	[53, 69]
Swimming medium	NaCl 0.5 g/L, peptone 10 g/L and agarose 3 g/L	[53, 69]
Twitching medium	NaCl 10 g/L, yeast extract 5 g/L, tryptone 10 g/L, and agar 15 g/L	[53, 69]
NGM medium	NaCl 3 g/L, tryptone 2.5 g/L, $MgSO_4$ 0.12 g/L, $CaCl_2$ 0.11 g/L, cholesterol 0.005 g/L,	[53]

FUDR (5-fluoro-2′-deoxyuridine) 0.1 g/L,
agar 17 g/L

#Add appropriate amount of DMSO, bakuchiol, or calycosin to the culture media as needed during the experiment.

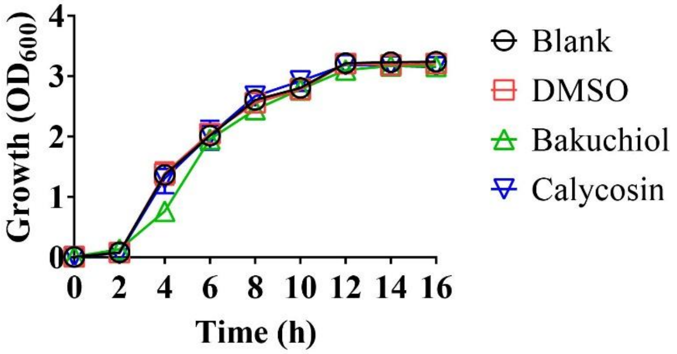

Figure S1. Effect of bakuchiol on the growth of *P. aeruginosa*. All data represent the results of at least three independent biological replicates. The error bars represent the standard deviations.

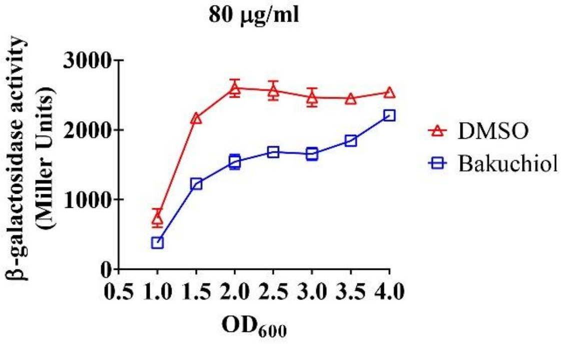

Figure S2. Effect of bakuchiol on *pqsA* expression in *P. aeruginosa* at different growth stages. All data represent the results of at least three independent biological replicates. The error bars represent the standard deviations.

References

53. Wang, S.; Feng, Y.; Han, X.; Cai, X.; Yang, L.; Liu, C.; Shen, L. Inhibition of Virulence Factors and Biofilm Formation by Wogonin Attenuates Pathogenicity of *Pseudomonas aeruginosa* PAO1 via Targeting *pqs* Quorum-Sensing System. *Int. J. Mol. Sci*. **2021**, *22*, 12699.

68. Essar, D. W.; Eberly, L.; Hadero, A.; Crawford, I. P. Identification and characterization of genes for a second anthranilate synthase in *Pseudomonas aeruginosa*: interchangeability of the two anthranilate synthases and evolutionary implications. *J. Bacteriol*. **1990**, *172*, 884-900.

69. Wang, S.; Yu, S.; Zhang, Z.; Wei, Q.; Yan, L.; Ai, G.; Liu, H.; Ma, L. Z.; Nojiri, H. Coordination of Swarming Motility, Biosurfactant Synthesis, and Biofilm Matrix Exopolysaccharide Production in *Pseudomonas aeruginosa*. *Appl. Environ. Microbiol*. **2014**, *80*, 6724-32.

71. Choi, K.-H.; Schweizer, H. P. mini-Tn7 insertion in bacteria with single *att*Tn7 sites: example *Pseudomonas aeruginosa*. *Nat. Protoc*. **2006**, *1*, 153-61.
77. Appanna, V. D.; Sarabhai, S.; Sharma, P.; Capalash, N. Ellagic Acid Derivatives from Terminalia chebula Retz. Downregulate the Expression of Quorum Sensing Genes to Attenuate *Pseudomonas aeruginosa* PAO1 Virulence. *PLoS. One*. **2013**, *8*, e53441.
78. Schäfer, A.; Tauch, A.; Jäger, W.; Kalinowski, J.; Thierbach, G.; Pühler, A. Small mobilizable multi-purpose cloning vectors derived from the Escherichia coli plasmids pK18 and pK19: selection of defined deletions in the chromosome of *Corynebacterium glutamicum*. *Gene*. **1994**, *145*, 69-73.
79. Kovach, M. E.; Elzer, P. H.; Hill, D. S.; Robertson, G. T.; Farris, M. A.; 2nd, R. M. R.; Peterson, K. M. Four new derivatives of the broad-host-range cloning vector pBBR1MCS, carrying different antibiotic-resistance cassettes. *Gene*. **1995**, *166*, 175-76.
80. Dennis, J. J.; Zylstra, G. J. Plasposons: modular self-cloning minitransposon derivatives for rapid genetic analysis of gram-negative bacterial genomes. *Appl. Environ. Microbiol*. **1998**, *64*, 2710-15.
81. Becher, A.; Schweizer, H. P. Integration-Proficient *Pseudomonas aeruginosa* Vectors for Isolation of Single-Copy Chromosomal *lacZ* and *lux* Gene Fusions. *BioTechniques*. **2018**, *29*, 948-52.
82. Choi, K.-H.; Mima, T.; Casart, Y.; Rholl, D.; Kumar, A.; Beacham, I. R.; Schweizer, H. P. Genetic Tools for Select-Agent-Compliant Manipulation of *Burkholderia pseudomallei*. *Appl. Environ. Microbiol*. **2008**, *74*, 1064-75.
